# UTX Responds to Nanotopography to Suppress Macrophage Inflammatory Response by Remodeling H3K27me3 Modification

**DOI:** 10.1002/advs.202505723

**Published:** 2025-05-19

**Authors:** Hengji Jia, He Zhang, Dingqiang Mo, Bo Xie, Hongdou Qiao, Tao Chen, Haoyue Song, Xinxin Xu, Sheng Yang

**Affiliations:** ^1^ College of Stomatology Chongqing Medical University Chongqing China; ^2^ Chongqing Key Laboratory of Oral Diseases and Biomedical Sciences China; ^3^ Chongqing Municipal Key Laboratory of Oral Biomedical Engineering of Higher Education China; ^4^ Chongqing Municipal Health Commission Key Laboratory of Oral Biomedical Engineering China

**Keywords:** histone methylation, macropahge, mechano‐immunology, nanotopography

## Abstract

Peri‐implantitis is the leading cause of implant failure, primarily due to weak defense at the implant–soft tissue interface, which disrupts the local immune microenvironment. As an integral part of this microenvironment, the implant–tissue interface plays a critical role in shaping immune cell function. Thus, engineering the surface topography of implants has emerged as a novel strategy for sustained immunomodulation following implantation. This study investigated the mechanical regulation of macrophage function by nanopatterned topographies. Titanium nanotubes (TNTs) surfaces reduce the expression of phosphorylated myosin light chain (pMLC) and promote the retention of the UTX histone methyltransferase in the nucleus. This process attenuates the enrichment of the repressive H3K27me3 histone marker at the *Abca1* gene locus, increasing *Abca1* expression and suppressing inflammation. This study reveals the mechanosensitivity of UTX and provides a new target for the development of therapeutic strategies that integrate mechanical signaling and immune modulation.

## Graphical abstract

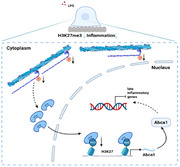

## 1 Introduction

Peri‐implantitis is a biological complication of peri‐implant tissues characterized by inflammation of the surrounding connective tissue and progressive alveolar bone loss.^[^ ^1^ ^]^ The clinical management of peri‐implantitis remains challenging because of its asymptomatic early stage and the limited long‐term efficacy of mechanical debridement and antibiotic therapy.^[^ ^2^, ^3^ ^]^ Therefore, achieving sustained immunomodulation of peri‐implant tissues is critical to mitigate peri‐implant inflammation.^[^ ^4^, ^5^ ^]^

As immune sentinels surrounding implants, macrophages constitute the first wave of innate immune cells recruited to the material‒tissue interface and play a central role in regulating inflammatory responses and remodeling the immune microenvironment.^[^ ^6^, ^7^ ^]^ After implantation surgery, macrophages are quickly recruited to the injury site, where they release inflammatory factors to clear debris, bacteria, and apoptotic cells, followed by the release of anti‐inflammatory factors to initiate tissue repair.^[^ ^8^, ^9^ ^]^ Persistent disruption of microenvironmental homeostasis, however, may induce macrophage hyperactivation, driving peri‐implant inflammatory progression that critically compromises functional implant integration. Emerging mechanoimmunological insights reveal that surface nanotopography is a promising strategy for long‐term macrophage modulation, leveraging advantages such as biomechanical stability and precise parameter control.^[^ ^10^, ^11^ ^]^ Recent studies have explored the mechanisms by which nanoscale morphology regulates macrophage inflammation, involving the aggregation of integrins,^[^ ^12^, ^13^ ^]^ remodeling of the cytoskeleton,^[^ ^14^ ^]^ and ion channels.^[^ ^15^ ^]^ Nevertheless, the potential mechanisms of mechanotransduction remain to be elucidated.

Epigenetics can regulate gene expression without altering the DNA sequence, determining cellular differentiation, function, and adaptability, ultimately influencing developmental processes and diseases.^[^ ^16^, ^17^, ^18^, ^19^ ^]^ Posttranslational modifications of histones, such as acetylation and methylation, represent principal epigenetic mechanisms. Specifically, trimethylation of histone H3 at lysine 27 (H3K27me3) serves as an important transcriptional repression marker that facilitates heterochromatin formation and establishes heritable gene silencing states.^[^ ^20^, ^21^, ^22^ ^]^ UTX, a specific demethylase for H3K27me3, erases methylation at this site, and its dysfunction has been implicated in various pathophysiological processes, including abnormal embryonic development,^[^ ^23^, ^24^, ^25^ ^]^ cellular senescence,^[^ ^26^ ^]^ and tumor immune evasion.^[^ ^25^, ^27^, ^28^ ^]^ Emerging evidence suggests that UTX is a central regulator of inflammatory cascades. For example, UTX knockout restores the blood‒spinal cord barrier, reduces inflammatory infiltration, and ultimately promotes neurological recovery.^[^ ^29^ ^]^ Additionally, UTX has been shown to enhance inflammation and DNA damage induced by palmitic acid, accelerating the progression of diabetic nephropathy.^[^ ^30^ ^]^ However, the responsiveness of UTX/H3K27me3 to nanotopography and the molecular mechanisms by which they regulate macrophage inflammation remain unclear.

In this study, we fabricated TNTs and demonstrated their superior anti‐inflammatory properties compared with those of pure titanium (pTi), as evidenced by the significant downregulation of LPS‐induced inflammatory gene transcription in macrophages. Mechanistically, TNTs weakened the contractility of macrophage myosin II and reduced H3K27me3 levels by enhancing the nuclear localization of the UTX histone demethylase, thereby inhibiting inflammatory gene transcription. Finally, RNA‐seq and CUT&Tag combined analyses revealed that the TNTs‐mediated reduction in H3K27me3 at the *Abca1* gene locus was directly correlated with *Abca1* transcriptional activation, establishing ABCA1 as a critical effector in the suppression of nanotopography‐driven inflammation. This study is the first to demonstrate the mechanosensitivity of UTX and its pivotal role in the regulation of macrophage inflammation mediated by topographical structures, providing molecular targets for the “mechanical‒epigenetic” pathway in inflammation regulation and laying a theoretical foundation for the development of immunomodulatory biomaterials.

## 2 Results

### 2.1 TNTs Inhibit the Macrophage Inflammatory Response Concomitant with a Reduction in Chromatin Condensation

**Figure 1:**
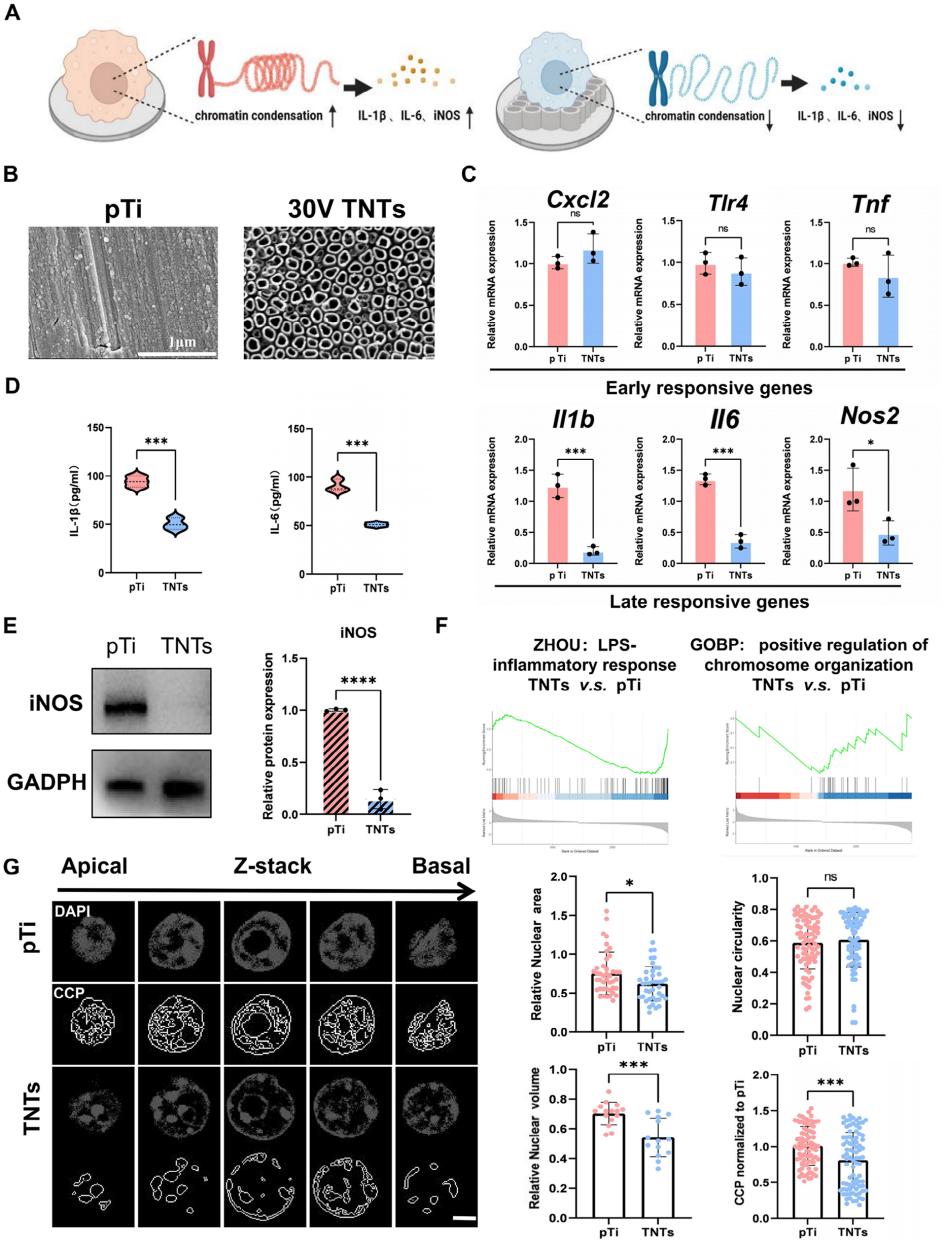
Titanium nanotubes inhibit the macrophage inflammatory response concomitant with a reduction in chromatin condensation. A) Diagrammatic illustration of nanotube‐mediated modulation of macrophage inflammatory activation, which correlates with a decrease in chromatin condensation. B) Scanning electron micrographs characterizing topographical variations among titanium substrates; scale bar: 1 µm. C) Comparative transcriptional profiling of proinflammatory mediators (immediate‐early and delayed‐response genes) in LPS‐stimulated macrophages adherent to titanium or nanotubes after 6 h of exposure. D,E) Relative secretion of the IL‐1β and IL‐6 late inflammatory factors (D) and relative protein expression of iNOS (E) in cells seeded on different titanium surfaces and treated with LPS for more than 6 h. F) GSEA showing significant differences in the enrichment of “LPS‐inflammatory response” and “positive regulation of chromatin organization” genes in cells seeded on different titanium surfaces and treated with LPS for more than 6 h. G) Representative images of nuclei from cells seeded on different titanium surfaces exposed to LPS, along with computational quantification of morphometric parameters, including nuclear planar spread, nuclear volumetric dimensions, sphericity indices, and the chromatin condensation index. These datasets were systematically acquired through analysis of 60–80 individual nuclei sampled across ≥20–30 randomized microscopic fields; scale bar: 2 µm. Other experimental measurements were calculated as the arithmetic mean derived from triplicate independent experimental replicates. All numerical outcomes are presented as the means ± standard deviations. Statistical significance thresholds were defined as *p < 0.05, **p < 0.01, and ***p < 0.001.

The surface morphology of the TNTs and pTi was characterized via SEM (**Figure** **1**B). Previous research by our group revealed that TNTs produced at 30 V strongly inhibit inflammation compared with pTi, providing a guide for conditions in subsequent studies.^[^ ^31^ ^]^ RAW264.7 cells were seeded on both TNTs and pTi surfaces and stimulated with LPS. The transcription of inflammatory genes was assessed by qRT‒PCR, which revealed that TNTs significantly downregulated the expression of the *II1b, II6*, and *Nos2* late inflammatory genes but had no significant effect on early inflammatory genes (Figure 1C). ELISA and Western blot analyses further confirmed the inhibition of late inflammation (Figure 1D,E). In summary, these data indicated that compared with pTi, TNTs effectively suppress macrophage inflammatory responses.

To elucidate the potential mechanisms by which TNTs inhibit macrophage inflammation, RNA sequencing and enrichment analysis were performed (Figure S1, Supporting Information). GSEA revealed that LPS‐induced inflammatory responses were lower in cells cultured on TNTs than in those cultured on pTi. Additionally, genes associated with the positive regulation of chromosome organization were significantly enriched in cells cultured on TNTs (Figure 1F). Previous studies have shown a close relationship between the transcription of late inflammatory genes and chromatin state.^[^ ^32^ ^]^ Therefore, we hypothesized that TNTs inhibit inflammatory gene transcription by remodeling chromatin states and assessed chromatin states on different surfaces (Figure 1G). Compared with macrophages on pTi, macrophages on TNTs exhibited a trend toward decreased chromatin condensation. Morphological analysis of the nuclei revealed that the cells in the TNTs group had smaller nuclear projection areas and volumes, although there was no significant difference in nuclear roundness (Figure 1G).

### 2.2 TNTs Suppress Inflammatory Gene Expression by Reducing H3K27me3 Levels

**Figure 2:**
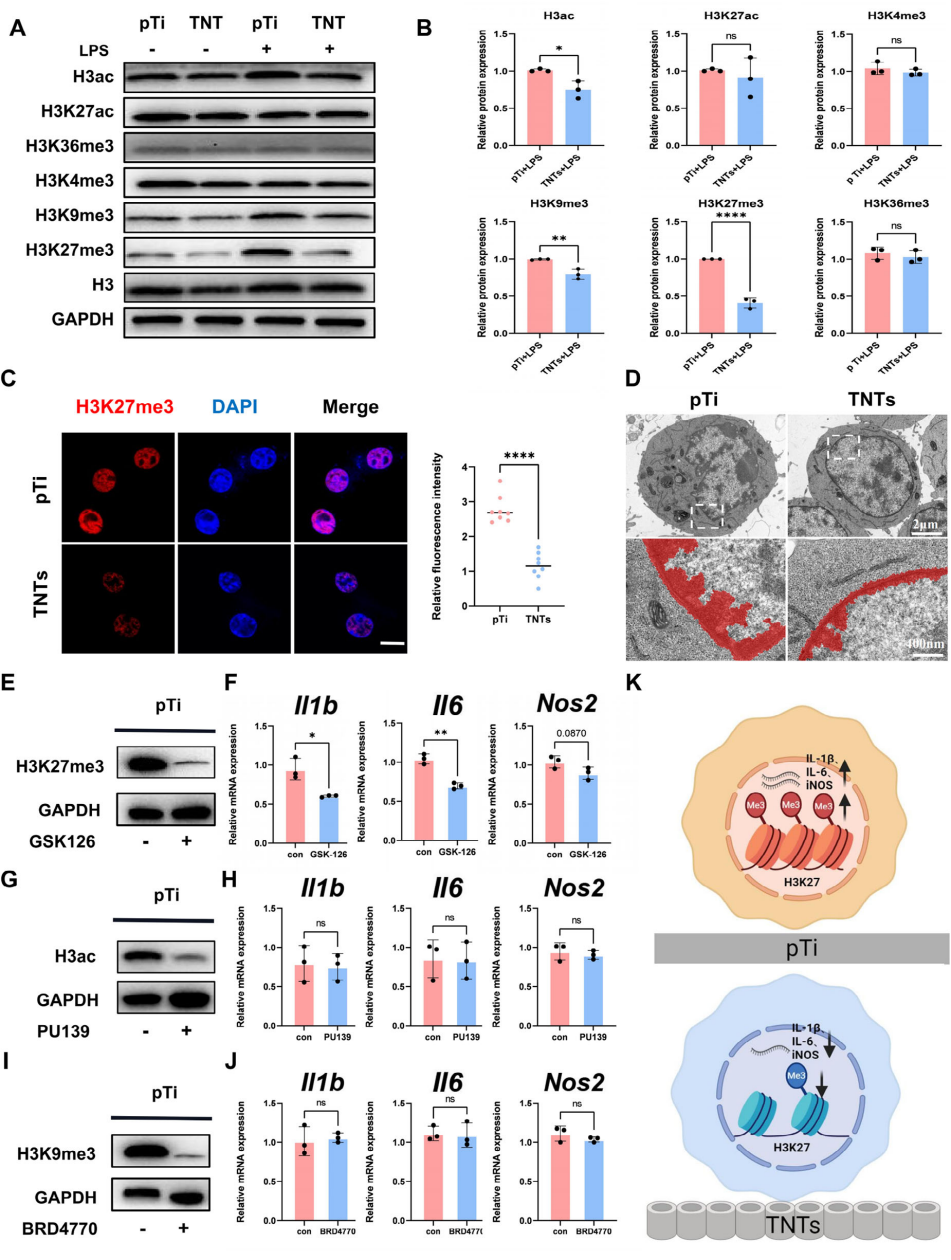
TNTs suppress inflammatory gene expression by reducing H3K27me3 levels. A,B) Expression and quantitative analysis of H3K27me3, H3K9me3, H3ac, H3K27ac, H3K4me3, and H3K36me3 in cells seeded on different titanium surfaces. C) Representative images and quantitative analysis of H3K27me3 protein levels in cells seeded on different titanium surfaces; scale bar: 5 µm. D) Representative transmission electron microscopy (TEM) images of heterochromatin in cells seeded on different titanium surfaces and treated with LPS for more than 6 h, with red areas indicating heterochromatin. E,G,I) Cells cultured on pTi were pretreated for 18 h with GSK‐126 (10 µM), PU139 (30 µM), BRD4770 (5 µM), or DMSO, followed by 6 h of LPS stimulation, and the relative protein expression levels of H3K27me3, H3K9me3, and H3ac were determined. F,H,J) Relative expression levels of inflammatory genes in macrophages on pTi after LPS stimulation with inhibitors or DMSO. (K) Schematic illustrating the impact of H3K27me3 on the transcription of inflammatory genes in cells seeded on different titanium surfaces. All numerical outcomes are presented as the means ± standard deviations. Statistical significance thresholds were defined as *p < 0.05, **p < 0.01, and ***p < 0.001.

Posttranslational modifications of histones, such as acetylation and methylation, are crucial mechanisms that influence chromatin states and play key roles in cell differentiation and function.^[^ ^16^, ^17^ ^]^ Therefore, we investigated whether TNTs inhibit the transcription of inflammatory genes in macrophages through histone posttranslational modifications. Western blot analysis revealed that cells cultured on TNTs presented lower levels of H3K27me3, H3K9me3, and H3ac, while the levels of H3K27ac, H3K4me3, and H3K36me3 modifications were similar (**Figure** **2**A). Statistical analysis (Figure 2B) revealed that the difference in H3K27me3 was the most pronounced. Immunofluorescence further confirmed the downward trend of H3K27me3 (Figure 2C). Compared with those cultured on pTi, the cells cultured on TNTs presented reduced heterochromatin thickness, with H3K27me3 serving as an important marker.

To investigate which histone modifications are responsible for the inhibitory effect of TNTs on inflammatory genes, epigenetic inhibitors were used to suppress the corresponding histone modifications. Compared with those in the control group, Western blot analysis revealed that GSK‐126, PU139, and BRD4770 significantly reduced the H3K27me3, H3ac, and H3K9me3 levels in the cells cultured on pTi (Figure 2E,G,I). Under LPS stimulation, macrophages seeded on pure titanium with GSK‐126 had lower mRNA expression levels of *Il1b* and *Il6*, while the mRNA expression levels of iNOS were not significantly different (Figure 2F). In contrast, the expression levels of the *II1b, II6*, and *Nos2* inflammatory genes did not change in cells treated with PU139 or BRD4770 (Figure 2H,J). Collectively, these findings indicated that TNTs modulate macrophage‐driven inflammatory activation through attenuation of H3K27me3 epigenetic modifications (Figure 2K).

### 2.3 TNTs Suppress H3K27me3 Levels and Inflammatory Gene Expression by Promoting UTX Nuclear Translocation

**Figure 3:**
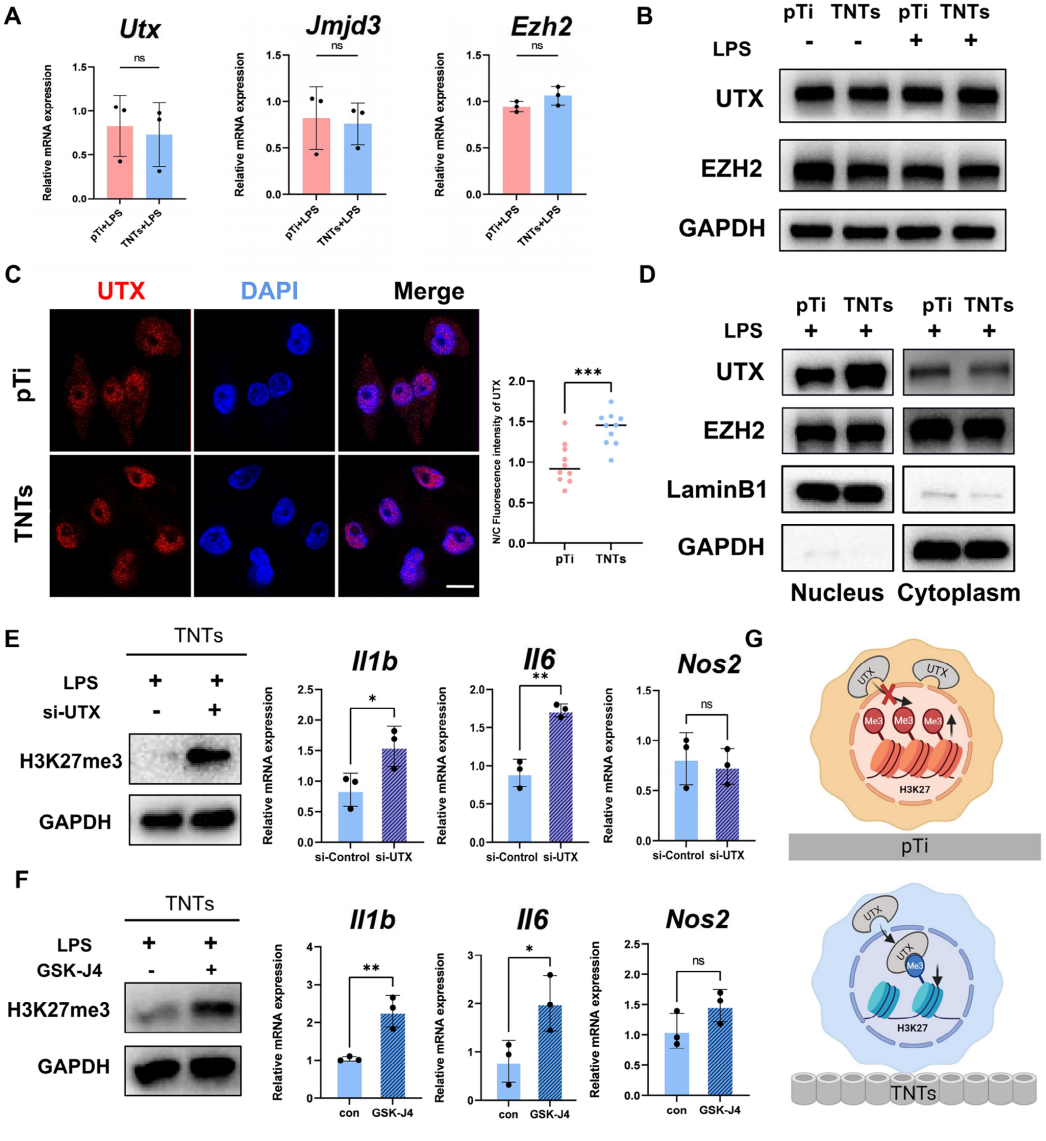
TNTs suppress H3K27me3 levels and inflammatory gene expression by promoting UTX nuclear translocation. A) Relative mRNA expression of *Utx*, *Jmjd3*, and *Ezh2* in cells seeded on different titanium surfaces and treated with LPS for more than 6 h. B) Relative protein expression of UTX and EZH2 in cells seeded on different titanium surfaces. C) Images of UTX (red) in cells seeded on different titanium surfaces and treated with LPS. Scale bar: 5 µm. The numerical statistical results of UTX were obtained from ≈10 cells selected from three independent samples. D) Representative Western blot images of UTX in the cytoplasm and nucleus of cells on pTi and TNTs after treatment with LPS. GAPDH served as an internal reference for cytoplasmic proteins, whereas LaminB1 served as an internal reference for nuclear proteins. E,F) After LPS treatment, the cells seeded on the nanotubes were also treated with GSK‐J4 (5 µM for 24 h) and si‐UTX (36 h) to assess the relative protein levels of H3K27me3 and the relative expression levels of inflammatory genes. G) Schematic illustrating the impact of UTX nuclear‐cytoplasmic localization on H3K27me3 in cells seeded on different titanium surfaces. All numerical outcomes are presented as the means ± standard deviations. Statistical significance thresholds were defined as *p < 0.05, **p < 0.01, and ***p < 0.001.

H3K27me3 is regulated by the EZH2 histone methyltransferase and the JMJD3 and UTX demethylases.^[^ ^33^ ^]^ To determine how TNTs decrease the level of H3K27me3 modification in macrophages, the expression levels of EZH2, JMJD3, and UTX were evaluated in cells from the TNTs and pTi groups. Because the gene expression and protein levels did not significantly change (**Figure** **3**A,B), we next investigated whether TNTs regulate H3K27me3 levels by promoting the nuclear translocation of methylation‐modifying enzymes. Immunofluorescence and Western blot analyses revealed that after LPS treatment, the nuclear translocation of UTX was greater in macrophages cultured on TNTs than in those cultured on pTi (Figure 3C,D).

To evaluate whether TNTs regulate the transcription of inflammatory genes by enhancing the nuclear localization of UTX, siRNA was used to knockdown UTX expression. The expression levels of *Il1b* and *Il6* were significantly increased in cells from the TNTs group (Figure 3E; Figure S2, Supporting Information), and the UTX inhibitor had the same effect (Figure 3F). Collectively, these experimental results demonstrated that TNTs regulate macrophage inflammatory responses by promoting the nuclear translocation of UTX (Figure 3G).

### 2.4 TNTs Regulate UTX Nuclear Translocation by Modulating Actomyosin Contractility

**Figure 4:**
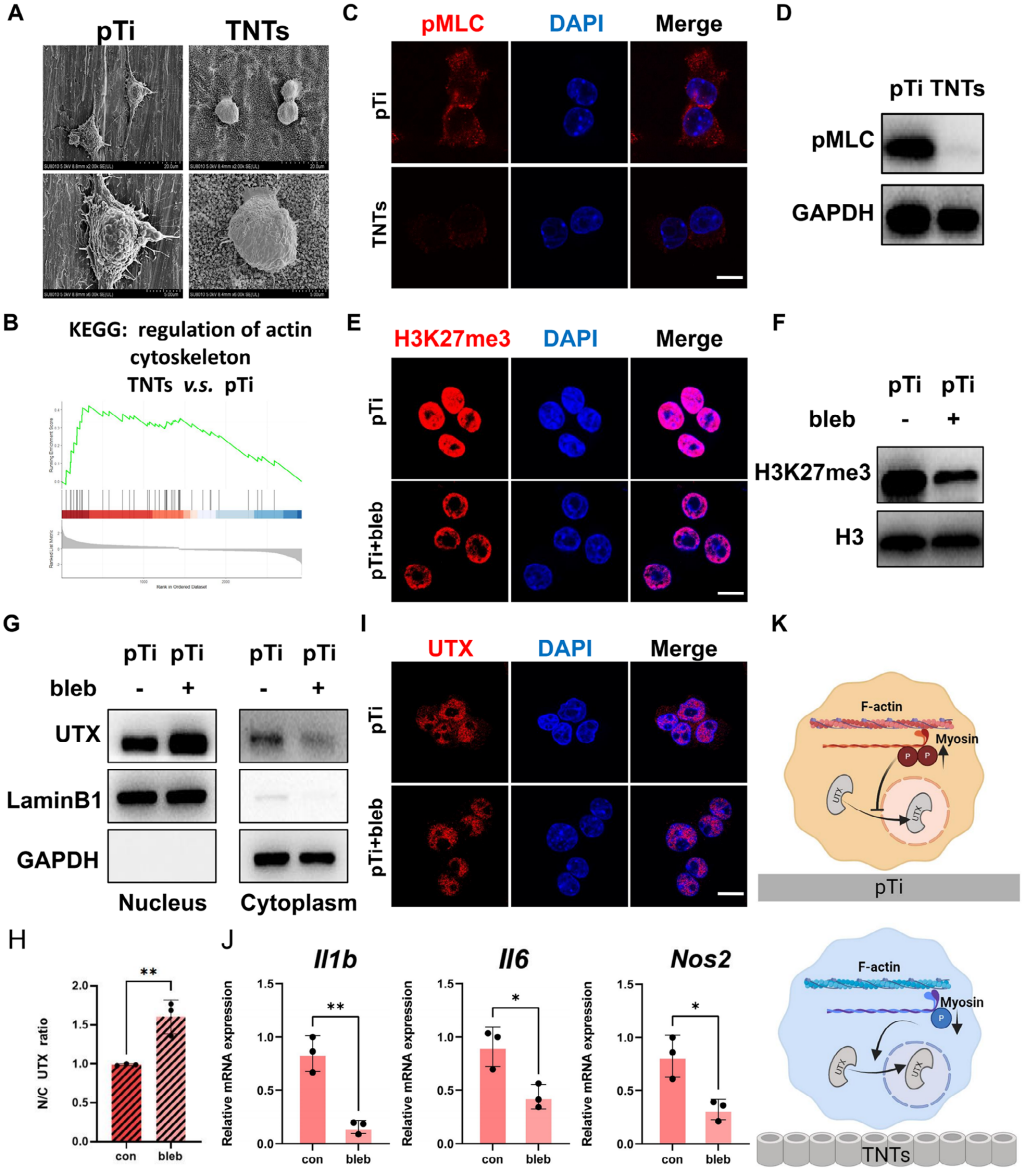
TNTs regulate UTX nuclear translocation by modulating actomyosin contractility. A) Cell morphology of macrophages treated with LPS and cultured on pTi and TNTs; scale bar as indicated in the figure. B) GSEA shows significant differences in the enrichment of “regulation of actin cytoskeleton” genes in cells adhered to different surfaces and treated with LPS. C,D) Representative images (C) and relative protein expression (D) of UTX (red) in cells adhered to different surfaces and treated with LPS; scale bar: 5 µm. E,F) Representative images (E) and relative protein expression (F) of H3K27me3 (red) in cells seeded on pure titanium and treated with or without blebbistatin for 1 h, followed by treatment with LPS for 6 h; scale bar: 5 µm. G–I) Relative protein levels, quantitative analysis, and representative images of cytoplasmic and nuclear UTX (G) in macrophages cultured on pTi and treated with or without blebbistatin for 1 h, followed by treatment with LPS for 6 h.; scale bar: 5 µm. GAPDH served as a loading control for cytoplasmic proteins, whereas LaminB1 was used as a loading control for nuclear proteins. J) Relative expression levels of inflammatory genes in cells adhered to pure titanium after LPS treatment with or without blebbistatin pretreatment. K) Schematic illustrating the effect of pMLC on the nuclear‒cytoplasmic localization of UTX in macrophages adhered to different surfaces. All numerical outcomes are presented as the means ± standard deviations. Statistical significance thresholds were defined as *p < 0.05, **p < 0.01, and ***p < 0.001.

SEM was employed to investigate how TNTs regulate the nuclear translocation of UTX. SEM images revealed that macrophages on pTi exhibited extensive cytoplasmic spreading and pronounced filopodial protrusions. Conversely, nanotube surfaces triggered cellular contraction into a spherical morphology accompanied by marked attenuation of filopodial development (**Figure** **4**A). Given that cell morphology is closely related to the cytoskeleton and that GSEA revealed significant enrichment of genes associated with microfilament cytoskeleton regulation in cells cultured on TNTs (Figure 4B), the cytoskeletal status of the cells seeded on the two surfaces was further assessed. We observed the actin polymerization (F‐actin expression) on different titanium surfaces. Despite significant changes in cell morphology and the arrangement of F‐actin, treatment with Cyto D did not result in notable changes in the levels of inflammatory genes or H3K27me3 modifications (Figure S3, Supporting Information).

However, immunofluorescence and Western blot analyses (Figure 4C,D) revealed that macrophages cultured on TNTs presented lower levels of pMLC, a recognized marker of actomyosin contractility.^[^ ^31^ ^]^To confirm that TNTs regulate H3K27me3 levels through actomyosin contractility, cells on pTi were treated with blebbistatin. As expected, blebbistatin promoted the nuclear translocation of UTX and suppressed the level of H3K27me3 (Figure 4E–I). Under inflammatory conditions induced by LPS, blebbistatin also reduced inflammatory gene expression in cells adhered to pure titanium (Figure 4J). These data suggest that TNTs promote UTX nuclear import by downregulating actomyosin contractility (Figure. 4K).

### 2.5 A Reduction in H3K27me3 Inhibits Macrophage Inflammation by Upregulating ABCA1

**Figure 5:**
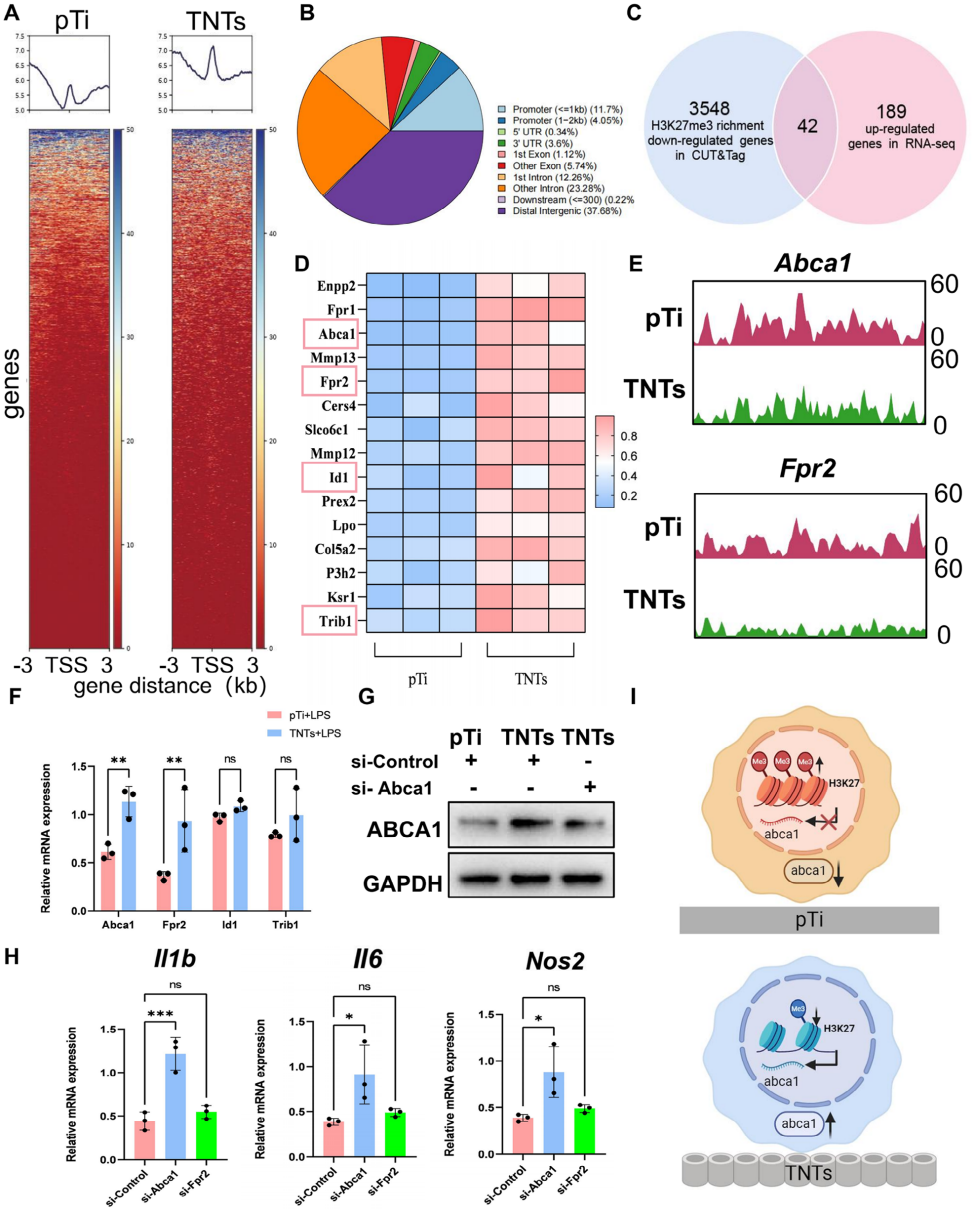
A reduction in H3K27me3 inhibits macrophage inflammation by upregulating ABCA1. A) Heatmap showing the genomic occupancy of H3K27me3 ±3 kb flanking TSSs in cells adhered to different surfaces after LPS stimulation, with genes displayed in descending order of signal intensity. B) Pie chart illustrating the distribution of H3K27me3 in annotated genomic regions across different titanium surfaces. C) Venn diagram showing the intersection of genes with downregulated H3K27me3 enrichment in CUT&Tag and upregulated genes identified via RNA‐seq in cells on TNTs. D) Among the 42 intersecting genes, the top 15 genes with differential expression levels are listed. E) Visualization of peak profiles for the *Abca1* and *Fpr2* genes. F) Relative mRNA expression of *Abca1*, *Fpr2*, *Id1*, and *Trib1* in cells adhered to different surfaces after LPS exposure. G) Relative protein expression levels of ABCA1 in cells adhered to different surfaces after LPS stimulation and exposure to si‐Control or si‐*Abca1*. H) Relative inflammatory gene expression in cells adhered to nanotubes after LPS stimulation and treatment with si‐Control, si‐*Abca1*, or si‐*Fpr2*. I) Schematic illustrating the impact of H3K27me3 on Abca1 transcription and protein levels in cells adhered to different surfaces. All numerical outcomes are presented as the means ± standard deviations. Statistical significance thresholds were defined as *p < 0.05, **p < 0.01, and ***p < 0.001.

To further explore how H3K27me3 regulates the expression of inflammatory genes, CUT&Tag analysis was performed. In contrast to the Western blot results, H3K27me3 peaks were higher near the transcription start sites (TSSs) in macrophages cultured on TNTs (**Figure** **5**A), suggesting that many of the enriched gene fragments may reside in the distal regions of the genes. Analysis of the genome‐wide distribution of H3K27me3 (Figure 5B) indicated that this histone modification is located primarily in distal intergenic regions and introns, with only 15.75% found in promoters (within ≤ 2 kb). This finding explains the discrepancy between the data shown in Figure 5A and the Western blot results. The analysis revealed that ≈3,548 gene regions were significantly downregulated and ≈522 gene regions were significantly upregulated.

Because H3K27me3 often represents transcriptional repression, the downstream target genes of H3K27me3 were investigated by intersecting the downregulated genes with the 189 upregulated DEGs identified by RNA‐seq analysis (Figure 5C), which identified 42 downstream genes. On the basis of the magnitude of the gene expression differences, the top 15 gene expression profiles are presented in Figure 5D. Studies have shown that *Abca1*, formyl peptide receptor 2 (*Fpr2*), DNA‐binding protein inhibitor ID‐1 (*Id1*), and tribbles pseudokinase 1 (*Trib1*) are closely associated with macrophage inflammation.^[^ ^34^, ^35^, ^36^, ^37^ ^]^ IGV visualization revealed a notable decrease in H3K27me3 peaks in the gene regions of *Abca1* and *Fpr2* (Figure 5E).

To assess whether *Abca1* and *Fpr2* mediate macrophage inflammatory responses, the expression levels of Abca1 and Fpr2 were measured in cells from the pTi and TNTs groups. Consistent with the transcriptomic results, TNTs significantly upregulated the expression of both *Abca1* and *Fpr2* (Figure 5F,G). Knockdown of *Abca1* led to the upregulation of *Il1b*, *Il6*, and *Nos2*, whereas knockdown of Fpr2 led to the upregulation of only *Il1b* expression (Figure 5H). These data demonstrated that downregulation of H3K27me3 promotes Abca1 expression and further inhibits macrophage inflammatory responses (Figure 5I).

### 2.6 TNTs Modification of Implant Surfaces can Alleviate Peri‐Implantitis

**Figure 6:**
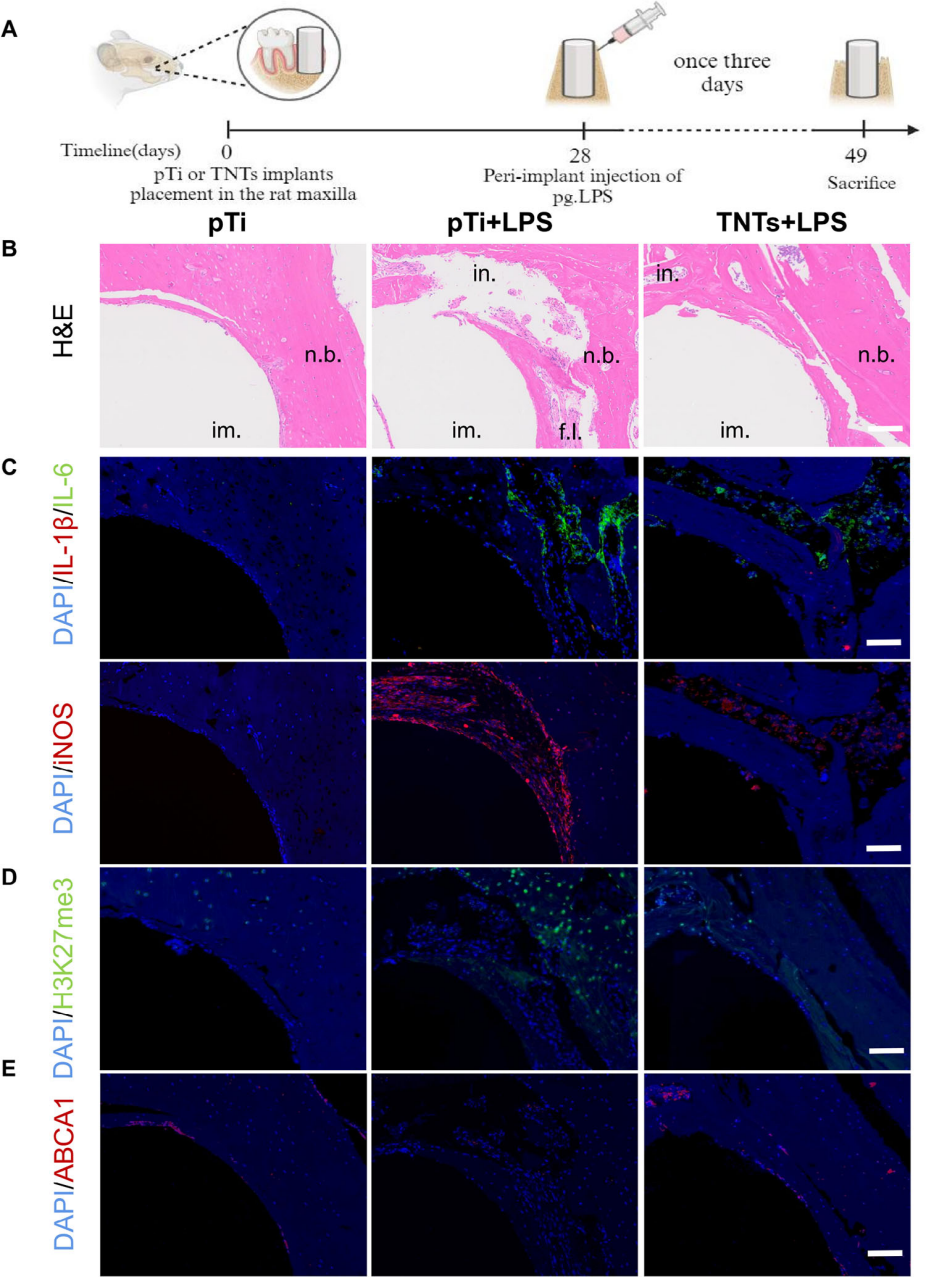
TNTs modification of implant surfaces alleviates peri‐implantitis. A) Schematic illustrating the construction of a rat peri‐implantitis model. B) Representative images of H&E staining of peri‐implant bone tissue. C–E) Representative immunofluorescence images of IL‐1β, IL‐6, iNOS, H3K27me3 and ABCA1 in peri‐implant bone tissues (im., implant; n.b., new bone; in., inflammatory; f.l., fibrous layer). Scale bar: 100 µm, n = 5.

A peri‐implantitis model was established by injecting LPS around the implants in the maxillary bones of rats (**Figure** **6**A), and the effects of TNTs on this condition were investigated. Histological analysis using H&E staining (Figure 6B) and TRAP staining (Figure S4, Supporting Information) revealed significant infiltration of inflammatory cells, bone tissue resorption, fibrous tissue regeneration, and osteoclast formation in the inflammation group. In contrast, the number of inflammatory cells and osteoclasts around TNTs was reduced, and enhanced bone tissue integration was observed.

Compared with those in the inflammation group, immunofluorescence analysis revealed fewer IL‐6‐, iNOS‐, H3K27me3‐positive and more ABCA1‐positive cells surrounding TNTs (Figure 6C–E), while the IL‐1β‐positive cells did not significantly differ between the two groups. In summary, TNTs exhibit favorable immunomodulatory properties and effectively suppress inflammation around implants.

## 3 Discussion

**Figure 7:**
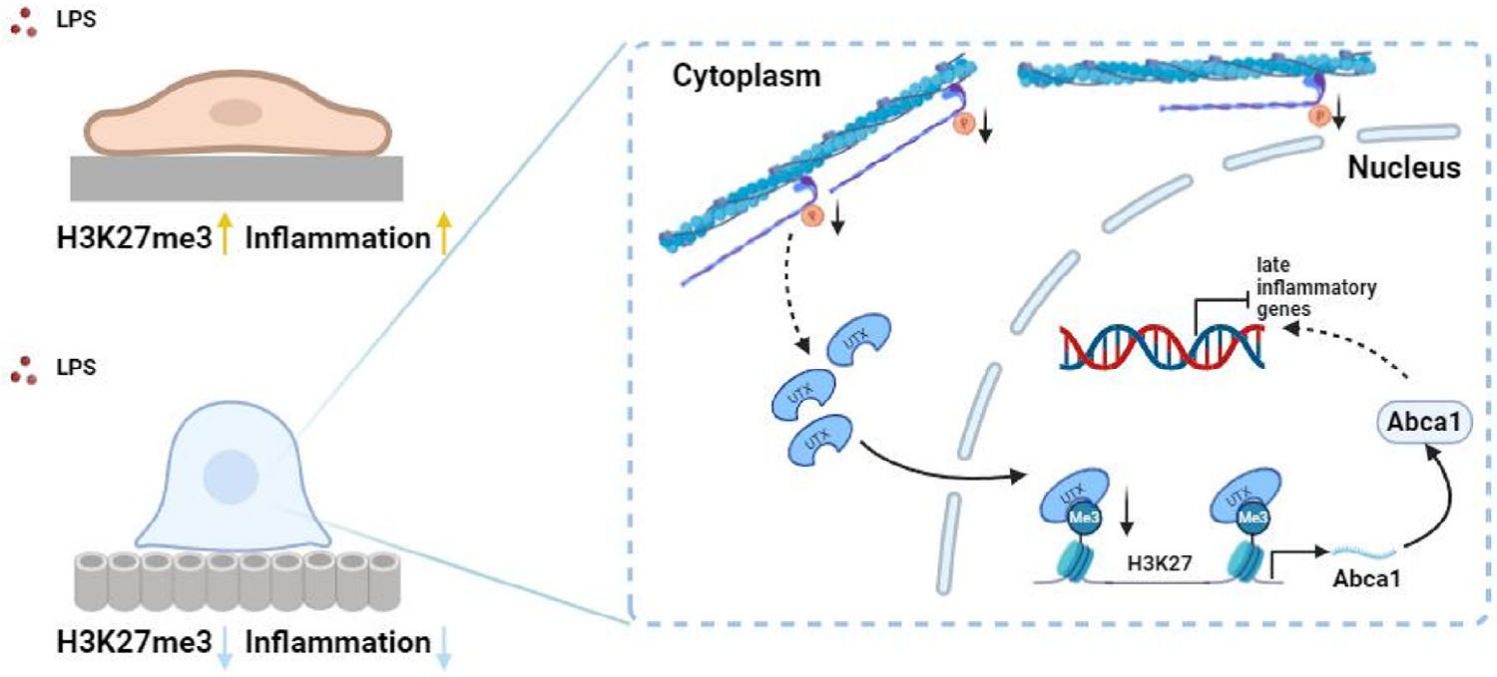
Schematic representation of how titanium nanotubes modulate macrophage H3K27me3‐mediated inflammatory responses through the nucleoplasmic localization of UTX. Compared with those on the pTi surface, macrophages on the TNTs surface present reduced myosin II contractility, which facilitates the nuclear translocation of the UTX. This reduction decreases the enrichment of the repressive H3K27me3 histone modification at the *Abca1* gene locus, which promotes the transcription and protein expression of ABCA1, thereby inhibiting the transcription of late inflammatory genes.

Peri‐implant inflammation is characterized by progressive tissue destruction due to an immune dysregulation response, primarily manifesting as persistent redness, exudation, and irreversible damage at the implant interface.^[^ ^3^ ^]^ Research has shown that macrophages accumulate significantly in inflammatory regions, exacerbating local oxidative stress responses through the secretion of proinflammatory factors such as IL‐1β and TNF‐α, ultimately leading to implant failure.^[^ ^2^, ^3^, ^4^ ^]^ In recent years, immunomodulation of the peri‐implant environment through material engineering has emerged as an effective strategy for maintaining long‐term implant stability. Compared with surface chemical modifications, surface topographical structures offer advantages that are direct, rapid, noninvasive, and durable.^[^ ^38^ ^]^ However, the unclear mechanisms of mechanotransduction severely limit the development of mechanically based immunomodulation strategies. In the present study, we constructed TNTs using an anodic oxidation method and reported that they reduce the contractility of macrophage myosin, promoting the nuclear translocation of the UTX histone demethylase. This action alleviates the transcriptional repression of H3K27me3 at the Abca1 gene locus, enhancing Abca1 expression while inhibiting the transcription of inflammatory genes. Finally, in vivo experiments demonstrated that TNTs play a positive role in suppressing peri‐implant inflammation. **Figure** **7** summarizes the mechanism of this pathway.

Epigenetic regulation plays a crucial role in the occurrence and development of inflammation.^[^ ^29^, ^30^ ^]^ For example, lactate serves as a primary fuel source for promoting H3K27ac, which enhances the expression of the Nr4a1 immune‐suppressive gene, thereby mediating the transcriptional repression of proinflammatory genes.^[^ ^39^ ^]^ However, the role of epigenetics in the regulation of inflammation mediated by nanotopological structures remains unclear. In the present study, macrophages cultured on TNTs presented increased nuclear localization of UTX and significantly lower H3K27me3 levels than those cultured on pTi, leading to the suppression of inflammatory gene expression. Research has indicated that H3K27me3 is involved in various cellular mechanotransduction pathways. For example, CytoD increases nuclear actin levels in mesenchymal stem cells, mediating the redistribution of H3K27me3,^[^ ^40^ ^]^ whereas mechanical stretching downregulates EZH2 protein expression, promoting H3K27me3‐mediated apoptosis in smooth muscle cells.^[^ ^41^ ^]^ Our study is the first study to demonstrate the mechanosensitivity of UTX, revealing a novel mechanotransduction pathway involving H3K27me3, which provides new molecular targets for mechanical immunomodulation. Notably, treatment of macrophages on TNTs with siRNA or UTX inhibitors increased the expression levels of inflammatory genes. In contrast to our results, previous studies have shown that UTX knockdown leads to the suppression of inflammation.^[^ ^42^, ^43^ ^]^ This discrepancy may be due to differences in cell types and culture substrates, thus warranting further investigation.

The present study also aimed to understand how UTX nuclear localization responds to TNTs. It is well established that cells react to external physical signals by altering their cytoskeletal structures.^[^ ^14^ ^]^ Moreover, histone‐modifying enzymes can undergo subcellular reorganization in response to certain mechanoreceptors.^[^ ^44^, ^45^ ^]^ The present study demonstrated that the contractility of actomyosin is crucial for mediating the subcellular localization of UTX in relation to TNTs and associated inflammatory gene expression. However, a direct regulatory relationship between pMLC and UTX was not observed. Further exploration using more advanced techniques, such as mass spectrometry and protein immunoprecipitation, will be necessary to investigate this relationship more comprehensively.

In the present study, we also explored how H3K27me3 regulates macrophage inflammation. Our results indicated that the downregulation of H3K27me3 levels leads to the suppression of inflammatory gene transcription, suggesting that TNTs mediate inflammation regulation by upregulating specific anti‐inflammatory factors associated with H3K27me3. To identify these regulatory factors, we utilized RNA‐seq and CUT&Tag analyses, which revealed that ABCA1 is a key bridging molecule in H3K27me3‐mediated regulation of macrophage inflammation. The ABCA1 protein is an important member of the ATP‐binding cassette (ABC) transporter superfamily, facilitating the efflux of free cholesterol and phospholipids to apolipoprotein A‐I (ApoA‐I), resulting in the formation of nascent high‐density lipoprotein (HDL). This reverse transport of cholesterol is crucial for clearing peripheral cholesterol from cells.^[^ ^46^, ^47^, ^48^ ^]^ Several studies have demonstrated the critical role of ABCA1 and ABCA1‐mediated cholesterol transport in inflammation. ABCA1 enhances cholesterol efflux and inhibits endoplasmic reticulum stress, reducing inflammatory infiltration and significantly preventing podocyte injury induced by high glucose and cholesterol.^[^ ^34^ ^]^ In Abca1^−/−^ macrophages, there is increased cholesterol accumulation in the endoplasmic reticulum, which leads to the deubiquitination of the NLRP3 inflammasome, thereby accelerating inflammation.^[^ ^49^ ^]^ Our study expands on these findings, suggesting that lipid metabolism, represented by ABCA1, may play a crucial role in peri‐implant inflammation, providing new molecular targets for the treatment of this condition.

## 4 Experimental Section

### Titanium Sample Preparation and Surface Characterization

Commercially pure titanium substrates (34/14 mm diameter × 1 mm thickness, 99.9% metallic purity; Baoji Titanium Group, China) underwent progressive surface refinement through mechanical abrasion using silicon carbide polishing discs (grit range: #320–#2000). Subsequent sonication in ethanol and deionized water yielded pTi surfaces. TiO2 nanotube arrays were synthesized via the anodic oxidation. In brief, polished titanium substrates were subjected to electrochemical treatment at 30 V for 60 min within a fluoride‐enriched electrolyte (composition: 10 g of NH4F in 500 mL of glycerol + 500 mL of dd H2O), followed by postanodization crystallization through thermal treatment (450 °C, 1 h, ambient atmosphere). All the samples were cleaned overnight in anhydrous ethanol using a shaker. After three 2‐h rinses with deionized water, the samples were dried and UV‐sterilized for 12 h before cell culture. The surface morphology was analyzed by SEM (Hitachi, Japan).

### Cell Culture

The RAW264.7 murine macrophage line was acquired from the National Collection of Authenticated Cell Cultures (NCACC, China). Cellular maintenance was conducted using Dulbecco's modified Eagle's medium (DMEM; Gibco, USA) supplemented with 10% fetal bovine serum (FBS; Gibco, USA) and a 1% antibiotic‐antimycotic cocktail (penicillin‒streptomycin; Solarbio, China). The cell populations were propagated under standardized conditions at 37 °C in a humidity‐controlled incubator containing 5% atmospheric CO₂.

### Drug Treatment

The cells were exposed to LPS (100 ng mL^−1^) (Sigma, China) for 6 h to induce proinflammatory activation.

The cells were treated with blebbistatin (40 µM, myosin II inhibitor) and cytochalasin D (5 µM, inhibitor of actin polymerization) for 7 h. For epigenetic studies, the cells were pretreated with GSK‐126 (10 µM), PU139 (30 µM), BRD4770 (5 µM), or GSK‐J4 (5 µM) for 18 h, followed by LPS treatment for 6 h.

### Quantitative Real‐Time PCR (qRT‒PCR)

Cellular total RNA was extracted via TRIzol reagent (Takara, Japan) and subsequently converted into cDNA via the PrimeScript Master Mix (Takara, Japan). Quantitative real‐time PCR was conducted with TB Green Premix Ex Taq II (Takara, Japan) on a CFX96 Real‐Time PCR Detection System (Bio‐Rad, USA). The primer sequences for the target genes are provided in Table S1 (Supporting Information), and glyceraldehyde‐3‐phosphate dehydrogenase (GAPDH) was utilized as the internal reference gene.

### Enzyme‐Linked Immunosorbent Assay (ELISA)

Cells adhered to different surfaces were exposed to LPS for 6 h. The supernatants were collected, and the concentrations of IL‐6 and IL‐1β were measured using ELISA kits according to the manufacturer's instructions (Meimian, China).

### Transcriptomic Analysis

Total RNA was isolated using RNAiso Plus reagent (Takara Bio, Japan) and subjected to transcriptomic profiling via RNA sequencing (NovelBio Biotechnology, Shanghai, China). Differentially expressed genes (DEGs) were identified through DESeq2 analysis (threshold: |log2‐fold change| >1 and false discovery rate [FDR]<0.05). The functional annotation of the DEGs was performed using the DAVID bioinformatics platform (https://david.ncifcrf.gov/) with Gene Ontology (GO) biological process and Kyoto Encyclopedia of Genes and Genomes (KEGG) pathway analyses. Gene set enrichment analysis (GSEA) was used to quantify pathway‐level dysregulation by evaluating coordinated expression changes in predefined gene sets.

### Morphological Observation of Macrophages

The samples were fixed in electron microscopy fixative for 1 h, followed by washing with PBS and immersion. Finally, cell morphology was observed using SEM.

### Transmission Electron Microscopy (TEM)

RAW264.7 cells were centrifuged at 1000 × g for 3 min and fixed with 4% glutaraldehyde at 4 °C for 24 h. The cells were then subjected to postfixation with 1% osmium tetroxide at 4 °C for 1 h. Dehydration was performed through a graded series of ethanol and acetone, followed by embedding in Epon 816 resin (Electron Microscopy Sciences, Hatfield, PA, USA). Ultrathin sections (70 nm) were prepared using a Leica ultramicrotome (Leica Microsystems, Buffalo Grove, IL, USA) and stained with uranyl acetate and lead citrate. TEM imaging was conducted via a transmission electron microscope (JEOL Ltd., Japan) operated at 120 kV.

### Western Blot Analysis

Whole‐cell lysates were extracted using RIPA lysis buffer (Beyotime, China) supplemented with protease and phosphatase inhibitors (Beyotime, China), followed by sonication for 15 min using an ultrasonic cell disruptor. Nuclear and cytoplasmic protein separation was performed using a Nuclear and Cytoplasmic Protein Extraction Kit (Beyotime, China) according to the manufacturer's protocol.

Proteins were resolved by SDS‒PAGE and transferred to PVDF membranes (Millipore, USA). The membranes were blocked with 5% nonfat dry milk for 2 h at room temperature and then incubated overnight at 4 °C with the following primary antibodies: iNOS (1:1000, CST, USA), H3ac (1:2000, Millipore, USA), H3K27ac (1:1000, Abcam, USA), H3K36me3 (1:1000, CST, USA), H3K4me3 (1:1000, CST, USA), H3K9me3 (1:1000, CST, USA), H3K27me3 (1:1000, CST, USA), H3 (1:1000, CST, USA), β‐actin (1:1000, CST, USA), GAPDH (1:1000, Proteintech, USA), EZH2 (1:1000, CST, USA), UTX (1:1000, CST, USA), laminB1 (1:1000, Abcam, USA), pMLC (1:1000, CST, USA), and ABCA1 (1:1000, CST, USA). The membranes were subsequently incubated with HRP‐conjugated secondary antibodies (1:5000; Proteintech, China) for 2 h at room temperature. After TBST washes, protein bands were visualized using enhanced chemiluminescence reagents (Millipore, USA) and imaged with a Bio‐Rad imaging system (Bio‐Rad, USA). Band intensities were quantified using Fiji.

### siRNA Transfection

Gene‐specific siRNA oligonucleotides targeting murine UTX, ABCA1, and FPR2 (sequences provided in Supplementary Table S2, Supporting Information) were procured from Tsingke Biotechnology (China).

For siRNA‐mediated gene silencing, RAW264.7 macrophages were cultured on titanium substrates until they reached 70% confluence. Transient transfection was performed using siRNA transfection reagent (SignaGen Laboratories, USA) according to the manufacturer's instructions, with a 36‐h incubation period prior to subsequent assays.

### Immunofluorescence Staining

Titanium‐adherent cells were fixed with 4% PFA for 15 min, washed three times with PBS, and permeabilized with 0.3% Triton X‐100 for 30 min. The samples were blocked with 5% donkey serum (Invitrogen, USA) for 1 h at RT. Primary antibody incubations were performed overnight at 4 °C with the following antibodies: H3K27me3 (1:400, CST, USA), UTX (1:400, CST, USA) and pMLC (1:400, CST, USA). After being washed with PBS, the samples were incubated with Alexa Fluor‐conjugated secondary antibodies (1:200, Invitrogen, USA) for 2 h at RT in the dark. Filamentous actin (F‐actin) was labelled using Alexa Fluor 488‐conjugated phalloidin (1:200, Invitrogen, USA). Nuclei were stained with DAPI (1:1000, Beyotime, China) for 2 min at RT. Immunofluorescence images were acquired using a confocal laser scanning microscope (LSCM; Leica Microsystems, Germany).

### Chromatin Condensation Parameter (CCP) Analysis

The cellular nuclei were examined via DAPI fluorescence labeling and visualized via an LSCM. For each experimental group, image acquisition included a minimum of six randomly selected observation areas. Nuclear segmentation was performed through manual image processing using FIJI, followed by computational analysis of the CCP through established algorithms referenced in prior research.^[^ ^50^ ^]^

### Cleavage Under Targets and Tagmentation (CUT&Tag) Sequencing

The ultraefficient universal CUT&Tag detection kit was purchased from Vazyme Biotech (Cat# TD903‐01; Nanjing, China). The experimental procedure was performed in strict accordance with the standard protocol. Briefly, biological replicates of RAW264.7 cells (1 × 10^5 cells per sample) were centrifuged and collected. After washing with wash buffer, the cells were incubated at room temperature for 10 min with magnetic beads precoated with lectin protein A. After the beads were collected, an overnight immunoconjugation reaction was performed at 4° C with a specific primary antibody (H3K27me3, 1:100). Following removal of the supernatant, the beads were incubated with a secondary antibody pretreated with Dig‐wash buffer at room temperature for 60 min. The beads were then collected and washed three times with wash buffer before a hyperactive pG‐Tn5/pA‐Tn5 transposase complex pretreated with Dig‐300 buffer was added, followed by incubation at room temperature for 1 h. After three washes with Dig‐300 buffer, 50 µL of labeling buffer was added, and the reaction for DNA labeling was conducted at 37 °C for 1 h. Subsequently, 100 µL of LA/B buffer, 5 µL of proteinase K (20 mg mL^−1^), and 20 µL of extraction magnetic beads were added, and the mixture was incubated at 55 °C for 10 min to terminate the labeling reaction. The samples were washed once with WA buffer (pretreated with anhydrous ethanol) and twice with WB buffer. After the supernatant was discarded, the DNA was eluted with 15 µL of elution buffer. Finally, the eluted DNA was subjected to PCR amplification and purification according to the kit instructions, followed by sequencing analysis.

### Animal Study

The present study received ethical approval from the Ethics Committee of the Affiliated Stomatological Hospital of Chongqing Medical University (Ethics No. CQHS‐REC‐2024 (LSNo.143)).

Sprague–Dawley (SD) rats (male, 250–300 g) were divided into the following three groups: 1) the pTi‐PBS group (implanted with pTi rods and injected with PBS); 2) the pTi group (implanted with pTi rods and injected with LPS); and 3) the TNTs group (implanted with TNTs rods and injected with LPS).

After the rats were anesthetized with 5 ml of tribromoethanol (Dowobio, China) via intraperitoneal injection, the first maxillary molar was surgically exposed. A hole was drilled into the mesial aspect of the bone, and a prepared titanium rod (diameter: 1 mm, length: 10 mm; Baoji Titanium Industry, China) was implanted, followed by suturing with 6–0 sutures.

One month later, LPS (1 mg mL^−1^, 20 µl) was injected at the surgical site every 3 days for a total of 7 injections, while PBS was injected into the control group. After the above procedures were completed, the animals were euthanized using carbon dioxide asphyxiation.

### Histological Detection

At the end of the experimental timeframe, the maxillary bones of the rats were collected and fixed in 4% paraformaldehyde for 24 h. The samples were then rinsed overnight and decalcified in 10% ethylenediaminetetraacetic acid (EDTA) for 30 days. After cleaning, dehydration, and embedding in paraffin blocks, the bones were sectioned into 5 µm slices.

For histological staining, the tissue sections were stained with hematoxylin and eosin (H & E; Solabrio, China) and an anti‐tartrate‐resistant acid phosphatase (TRAP) staining kit (Solabrio, China) according to the manufacturer's instructions.

The sections were washed, permeabilized, and blocked, followed by overnight incubation at 4 °C with the following primary antibodies: rat iNOS (1:100, Servicebio), IL‐1β (1:8000, Servicebio), IL‐6 (1:2000, Servicebio), H3K27me3 (1:1000, CST) and ABCA1 (1:200, Abcam, USA). The sections were then incubated with the following secondary antibodies: Alexa Fluor‐conjugated donkey anti‐rabbit IgG (1:200, Yeasen) and Alexa Fluor 594‐conjugated donkey anti‐mouse IgG (34112ES60, 1:200, Yeasen).

### Statistical Analysis

The data are presented as means ± SDs. All the statistical analyses were performed with GraphPad Prism 9 software (GraphPad, USA). Equal variance data were analyzed by one‐way ANOVA or Student's t test. Statistical significance was considered at *p < 0.05, **p < 0.01, and ***p < 0.001.

## Conflict of Interest

The authors declare no conflict of interest.

## Supporting information



Supporting Information

## Data Availability

The data that support the findings of this study are available from the corresponding author upon reasonable request.
