# Supplementary material for: UTX Responds to Nanotopography to Suppress Macrophage Inflammatory Response by Remodeling H3K27me3 Modification
Source: Adv Sci (Weinh). 2025 May 19;12(29):e05723. doi: 10.1002/advs.202505723 (PMC12362724; doi:10.1002/advs.202505723)
Supplement: Supplementary file 1 — Supporting Information [file ADVS-12-e05723-s001.docx]

**Supplementary Materials for**

**UTX Responds to Nanotopography to Suppress Macrophage Inflammatory Response by Remodeling H3K27me3 Modification**

Hengji Jia^1^，He Zhang^1,2,3,4*^，Dingqiang Mo^1^，Bo Xie^1^，Hongdou Qiao^1^，Tao Chen^1,2,3,4^，Haoyue Song^1^，Xinxin Xu^1,2,3,4*^，Sheng Yang^1,2,3,4*^

1, College of Stomatology, Chongqing Medical University;

2, Chongqing Key Laboratory of Oral Diseases and Biomedical Sciences;

3, Chongqing Municipal Key Laboratory of Oral Biomedical Engineering of Higher Education;

4, Chongqing Municipal Health Commission Key Laboratory of Oral Biomedical Engineering.

***Corresponding author**

E-mail addresses: [kqzhanghe@hospital.cqmu.edu.cn](mailto:kqzhanghe@hospital.cqmu.edu.cn) (H.Z.); 501638@hospital.cqmu.edu.cn (X.X.); [500283@cqmu.edu.cn](mailto:500283@cqmu.edu.cn) (S.Y.)


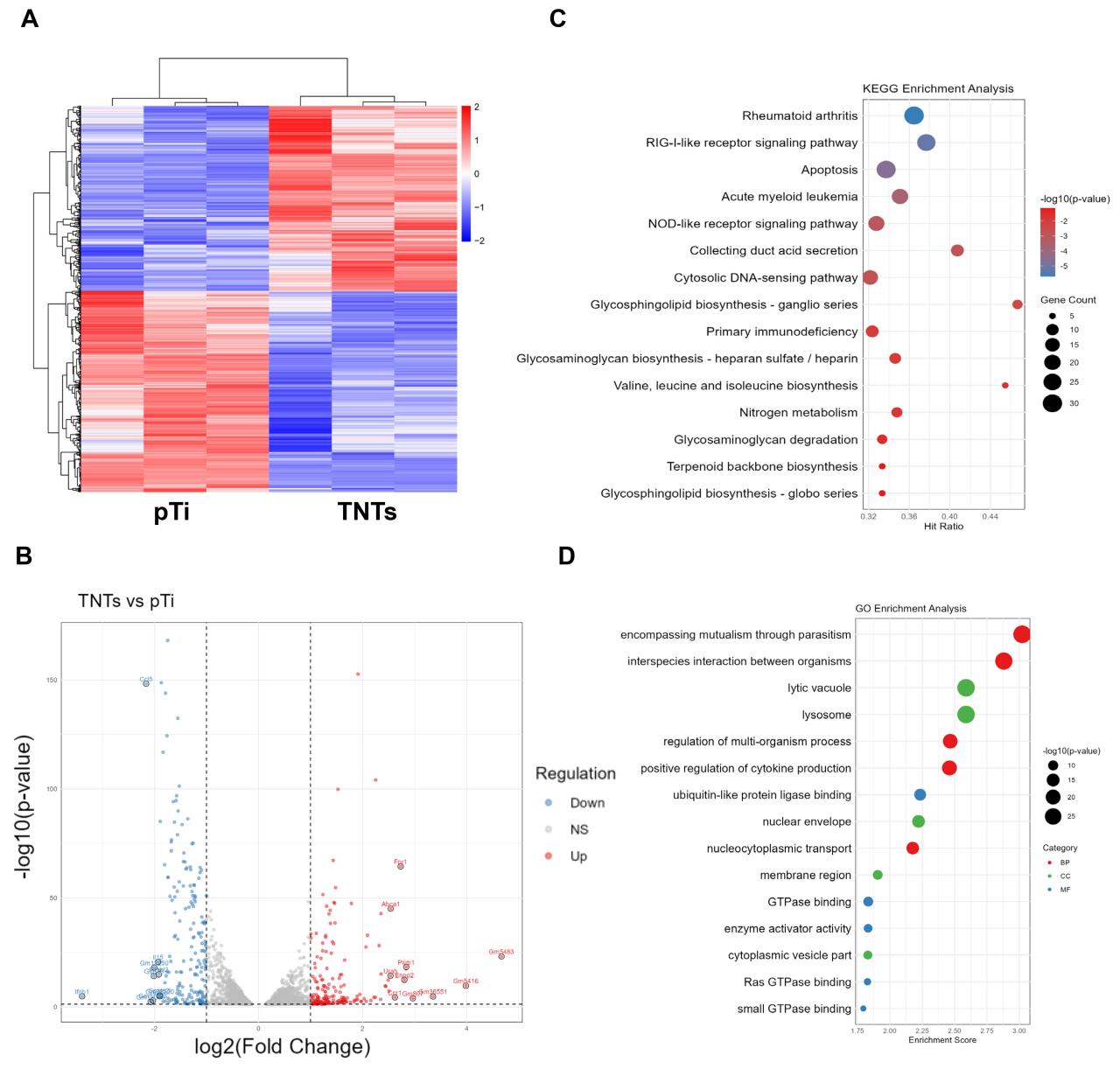


**Figure S1. Macrophage transcriptome analysis on pTi and TNTs.** (A) Differentially expressed genes (DEGs) between the pTi and TNTs groups (n = 3 per group) after LPS treatment. Red indicates upregulated expression levels, while blue indicates downregulated expression levels. (B) Volcano plot of the DEGs. (C) KEGG analysis showing the top 15 significantly enriched pathways. (D) Bubble plot of GO enrichment analysis.

**Fig S2.** Western blotting analysis of UTX expression in macrophages treated with UTX siRNA or nontargeting control for 36 hours. The data are presented as the mean ± SD of three biological replicates. ***p<0.001.

**Fig S3.** (A)Representative images of F-actin and DAPI staining in macrophages cultured on pTi and TNTs and stimulated with LPS for 6 hours. (B)Western blotting analysis of H3K27me3 in macrophages cultured on pTi and treated with cytoD. (C) Relative expression levels of inflammatory genes in macrophages on pTi after LPS stimulation with cytoD or PBS. Scale bar: 5 μm，ns：no significance（p＞0.05）.

**Fig S4.** Representative images of TRAP staining of peri-implant bone tissue.Scale bar: 100 μm, n = 5.

**Table S1. Primers used in qRT-PCR.**

| **Target** | **Primers** |
| --- | --- |
| *Il1b* | Forward 5’- TTGAAGTTGACGGACCCCA  Reverse 5’- GAGTGATACTGCCTGCCTGAAG |
| *Il6* | Forward 5’- GTTGCCTTCTTGGGACTGATG  Reverse 5’- TTGGGAGTGGTATCCTCTGTGA |
| *Nos2* | Forward 5’- TGGAGCGAGTTGTGGATTGT  Reverse 5’- TCTCTGCCTATCCGTCTCGTC |
| *CXCL2* | Forward 5’- CCAACCACCAGGCTACAGG  Reverse 5’- GCGTCACACTCAAGCTCTG |
| *Tlr4* | Forward 5’- ATGGCATGGCTTACACCACC  Reverse 5’- GAGGCCAATTTTGTCTCCACA |
| *TNF* | Forward 5’- CAGGCGGTGCCTATGTCTC  Reverse 5’- CGATCACCCCGAAGTTCAGTAG |
| *Gapdh* | Forward 5’-TGAGGTGACCGCATCTTCTTG  Reverse 5’- TGGTAACCAGGCGTCCGATA |
| *EZH2* | Forward 5’- CTTTGCTAAGAGGGCTATCCAG  Reverse 5’- CCACATACTTCAGGGCATCAG |
| *UTX* | Forward 5’- GCACCCACTCTACCTCATAACC  Reverse 5’- ACCTGCCAAATGTGAACTCG |
| *JMJD3* | Forward 5’- TATTCCTGTTTACCGCTTCGTG  Reverse 5’- CGTTCTTCACCTCGTTCCACT |
| *Abca1* | Forward 5’- GCACAATTTGTCCCTTCCAAGAT  Reverse 5’- CCGCATCACCAAGCTGAATAATT |
| *Fpr2* | Forward 5’- CACAGGAACCGAAGAGTGTAAGA  Reverse 5’- AACCACCACTTCTGATCCATTCA |
| *Id1* | Forward 5’- TCTTGTTCTCTTCCCACACTCTG  Reverse 5’- CGACAGACCAAGTACCACCTC |
| *Trib1* | Forward 5’- ACTACCTGCTGCTACCCCTA  Reverse 5’- CTTGCTCTCACCAAGGAGCA |

**Table S2. si-RNA sequences.**

| **Target** | **Sequences** |
| --- | --- |
| *UTX* | sense（5'-3'）GCCUAUGGAUGCUUUGCAA(dT)(dT)  anti-sense（5'-3'）UUGCAAAGCAUCCAUAGGC(dT)(dT) |
| *Abca1* | sense（5'-3'）GACGGGAUCUGAGAAGAAA(dT)(dT)  anti-sense（5'-3'）UUUCUUCUCAGAUCCCGUC(dT)(dT) |
| *Fpr2* | sense（5'-3'）GACUUUCGUGAGAGAUUUA(dT)(dT)  anti-sense（5'-3'）UAAAUCUCUCACGAAAGUC(dT)(dT) |
